# A Scoping Review of Health Outcomes Examined in Randomized Controlled Trials Using Guided Imagery

**DOI:** 10.1097/pp9.0000000000000010

**Published:** 2017-12-11

**Authors:** Peter R. Giacobbi, Jonathan Stewart, Keeley Chaffee, Anna-Marie Jaeschke, Meagan Stabler, George A. Kelley

**Affiliations:** a Department of Sport Sciences, West Virginia University, Morgantown, W. Va.; b Department of Social and Behavioral Sciences, West Virginia University, Morgantown, W. Va.; c Geisel School of Medicine, Dartmouth Institute for Health Policy and Clinical Practice, Dartmouth, Lebanon, N.H.; d Department of Biostatistics, West Virginia University, Morgantown, W. Va.

**Keywords:** guided imagery, randomized controlled trials, scoping review

## Abstract

**Introduction:**

Guided imagery involves the controlled visualization of detailed mental images. This integrative health technique is used for healing, health maintenance, or the treatment of specific conditions. Guided imagery is an integral part of mindfulness meditation, hypnosis, and various relaxation exercises. However, evidence to support the widespread use and dissemination of guided imagery interventions has been lacking. The purposes of this scoping review were to document the scope of health outcomes and disease processes examined by guided imagery researchers and the journal outlets where this work has been published. Secondary purposes were to review the efficacy of guided imagery, risk of bias from studies published in selected integrative health journals, and gain feedback from clinicians in a practiced-based research network about potential barriers for use in clinical settings.

**Methods:**

Ten bibliographic databases were searched for randomized controlled trials (RCTs) published between 1960 and 2013 that included adult participants. Descriptive and analytic methods were employed to document the journal outlets, diseases, and health outcomes investigated.

**Results:**

Three hundred twenty RCTs that included more than 17,979 adult participants were reviewed. The published studies appeared in 216 peer-reviewed journals from diverse disciplines largely representing psychology, the sport sciences, rehabilitation, nursing, and medicine. Major outcomes observed were coping with pain, stroke recovery, anxiety, coping with stress, and sport skills. Practitioner feedback from the practiced-based research network revealed some interest but skepticism and time constraints were discussed as barriers.

**Conclusions:**

Ongoing research and creative dissemination techniques are warranted.

## Abstract 2

Supplemental Digital Content is available in the text.

## Introduction

Guided imagery is a quasi-perceptual, multisensory, and conscious experience that may resemble the actual perception of a scene or event but occurs in the absence-specific stimuli.^[1,2]^ Also known as visualization or mental rehearsal, this technique is used for healing, health maintenance, or the treatment of specific conditions.^[1,2]^ Recent data from the United States National Center for Health Statistics shows that approximately 5 million adults reported using guided imagery primarily for stress reduction but also to address specific health complaints.^[1]^ Guided imagery is often used during mindfulness meditation, hypnosis, and various relaxation exercises since each of these techniques involve the creation and controlled visualization of mental images.^[3,4]^

Similar to other integrative health techniques, there appears to be significant public and scientific interest in guided imagery. For example, an Internet search using Google on March 29, 2017, and the term “guided imagery,” yielded more than 9.5 million results derived from numerous clinics and publications. These citations focused on ways to use imagery in various contexts (e.g., sport, rehabilitation, and self-help), its use as a relaxation technique, and media coverage of scientific findings by guided imagery researchers. Likewise, systematic reviews and meta-analyses of randomized controlled trials (RCTs) have been reported on the effects of imagery in the performance of motor, strength, and cognitive tasks,^[5]^ as a way to alter pain perceptions,^[6]^ its use by nursing practitioners for symptom management,^[7]^ and as part of other psychosocial treatments for depression and anxiety among cancer patients.^[8]^ More recently, researchers have shown that guided mental imagery can help individuals increase physical activity,^[9,10]^ modify food consumption and cravings,^[11–13]^ and cope with stress.^[14,15]^ Finally, neuroscientists have also extensively studied the cognitive processes associated with imagery for problem solving, speech, motor function, and memory.^[16]^

Although the observations above provide insight into some of the issues studied by guided imagery researchers, it is likely that scientists from other disciplines are investigating the impact of this cognitive technique on a wide-range of health outcomes. Documenting the outcomes of guided imagery interventions and the journal outlets where this work is published could inform future research on this topic by identifying gaps particularly in preventive behavior change research. Therefore, the first purpose of this study was to provide a descriptive review of the outcomes studied in RCTs from 1960 to 2013 that tested the impact of guided imagery, including the publication outlets where these works were published.^[17]^ A second purpose was to evaluate the efficacy of guided imagery by reviewing the methods and results of RCTs published in selected integrative health journals. Finally, a third purpose was to consult with clinicians about the study results to understand possible barriers to implementation in clinical settings (effectiveness). Scoping reviews are ideally suited to address all 3 purposes since they can map the range, extent, and nature of research activity and may inform future research, including quantitative reviews.^[18,19]^ Importantly, scoping reviews differ from systematic reviews in the wider potential breadth of research questions that can be addressed as well as an emphasis on the narrative integration of research evidence.^[19]^

## Methods

### Overview

This scoping review represents a secondary analysis from a previously published study.^[20]^ The steps for the current scoping review included the following: (1) identification of the research questions; (2) identification of relevant studies; (3) study selection; (4) charting of the data; (5) collating, summarizing, and reporting the results; and (6) receiving practitioner feedback from a practice-based research network (PBRN) in West Virginia.^[19]^ The last step, as suggested by Levac et al.^[19]^ was conducted for the purpose of encouraging wider dissemination and use of guided imagery. This was accomplished by sharing a 1-page summary of our findings with a state wide PBRN. For the purposes of this review, we were especially interested in clinicians’ views about the potential for wider dissemination and translation of guided imagery in clinical settings. Finally, we chose to review studies dating back to 1960 to gain a comprehensive assessment of randomized trials evaluating the impact of guided imagery, which is in line with the strengths of a scoping review.

### Study Identification and Data Sources

Citations were retrieved from 10 electronic bibliographic databases (Academic Search Complete, Medline from Ebscohost, PsycInfo, Scopus, SPORTDiscus, Cochrane Central Register of Controlled Clinical Trials, Cumulative Index to Nursing and Allied Health Literature, Physiotherapy Evidence Database, Web of Science, and ERIC). Keywords included, but were not limited to, random, mental imagery, guided imagery, visualization, and relaxation. In addition, the terms randomly and randomized were used to increase retrieval of studies that met our inclusion criteria. Furthermore, a range of health terms and disease processes were included in this search. A Health Sciences librarian with experience conducting systematic reviews conducted all searches in consultation with the research team. An example of the search strategy for one of the database searches is included in Supplementary File 1 (see **Supplemental Digital Content 1**, http://links.lww.com/PP9/A0).

### Identification and Selection of Relevant Studies

For purpose 1 of the scoping review, the inclusion criteria included the following: (1) RCTs with at least 1 comparison condition; (2) adult participants 18 years of age and older; (3) use of guided imagery as a sole or partial intervention strategy; and (4) publications in English from January 1, 1960, to June 1, 2013. Studies were selected by 3 authors (J.S., A.M.J., and M.S.) who independently reviewed all studies in consultation with the 2 senior investigators (P.G. and G.A.K.).

To address the second purpose of this study, we focused on 4 journals in integrative, alternative, and complementary health: *Journal of Complementary and Alternative Medicine*, *Applied Psychophysiology and Biofeedback*, *Contemporary Hypnosis*, and *Alternative Therapies*. These journals and publications were chosen because they included physical and psychological outcomes plus the use of subjective (e.g., survey ratings) and objective measures (e.g., blood loss, muscular activation). In addition to coding outcomes of these studies, we evaluated risk of bias in these studies using the original Cochrane Risk of Bias tool, an instrument that evaluates 6 sources of potential bias in RCTs:^[21]^ (1) random sequence generation (selection bias); (2) allocation concealment (selection bias); (3) blinding of participants, personnel, and outcome assessment (performance and detection bias); (4) incomplete outcome data (attrition bias); (5) selective reporting (reporting bias); and (6) other sources of bias. All items reported were rated as “low,” “high,” or “unclear” risk. In addition, a separate, self-developed, risk of bias coding category for blinding was also included that allowed us to evaluate whether studies blinded research personnel, participants, or both. This 5-item coding scheme included the following: 0 = No blinding; 1 = Blinding of participants only; 2 = Blinding of testers only; 3 = Double blind (participants and testers); and 4 = Not indicated.

### Data Charting and Synthesis

A codebook was developed by the first and third author working closely with a senior investigator (G.A.K.). The broad categories of variables coded included the following: (1) journal publication and year; (2) study author(s); (3) study design; and (4) outcomes measured. The third author pilot tested the codebook during the preliminary stages of the investigation. Subsequently, 2 authors (J.S. and M.S.) independently coded, with input from the first author, all studies that met the inclusion criteria.

Study outcomes were charted and synthesized in 3 steps. First, 2 authors (J.S. and M.S.) read each abstract and documented the specific outcomes being investigated from terms used in the study titles, abstracts, and key words. This process allowed the first 2 authors to observe thematic similarities in the outcomes, which then lead to the development of a coding template. This template was then used in a second round of coding by the same 2 authors to qualitatively describe the outcomes observed. A list of outcomes was developed by the first 2 authors (P.G. and J.S.) and each outcome was given a dummy code. The outcome frequencies were then calculated among all the included studies using the frequency command in IBM’s Statistical Package for the Social Sciences (IBM SPSS).

Finally, we consulted the West Virginia Practice Based Research Network to address the third purpose of this study and follow Levac et al.^[19]^ sixth criterion for scoping reviews. This network, funded by a Clinical and Translational Sciences Institute, includes 73 clinical partners representing medicine, behavioral health and pharmacy. This was accomplished by sending an e-mail to members of the WV PBRN inviting them to respond to a Qualtrics survey (see **Supplemental Digital Content 2**, http://links.lww.com/PP9/A1). The Survey asked PBRN members 2 questions: (1) Please share your general thoughts about the potential use of guided imagery in your clinical practice and (2) Please describe any potential barriers or facilitators for the use of guided imagery in your clinical practice considering that this cognitive technique can be delivered remotely or in-person.

## RESULTS

Of the 1,935 publications screened, 320 RCTs that included more than 17,979 adult participants met the criteria for inclusion. The exact sample sizes for several investigations were unclear so a precise sum of participants was not possible. The published studies appeared in 216 peer-reviewed journals from a range of disciplines and topic areas. Table 1 shows the journal outlets where 3 or more publications appeared while Table 2 includes the outcomes reported in these articles.

**TABLE 1.**
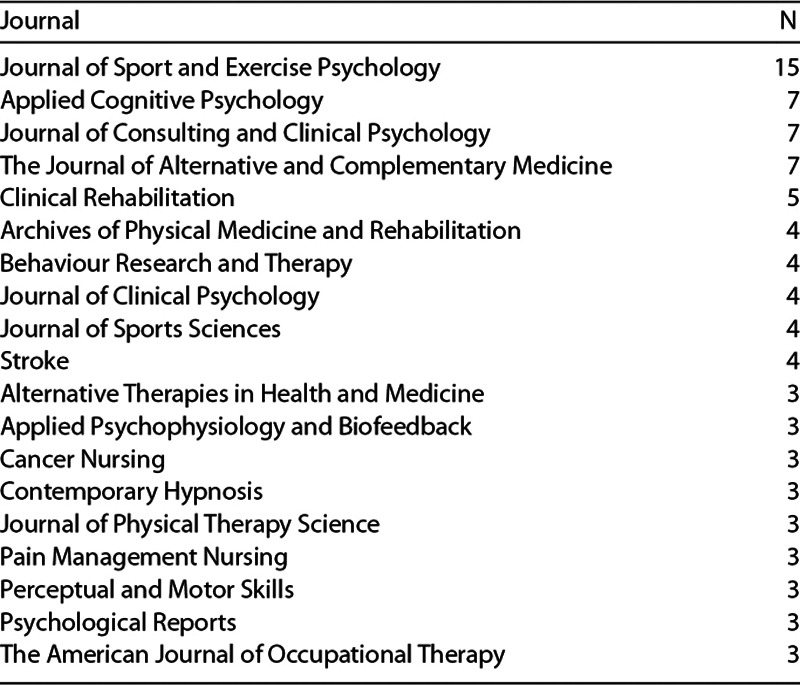
Journals with 3 or More Publications

**TABLE 2.**
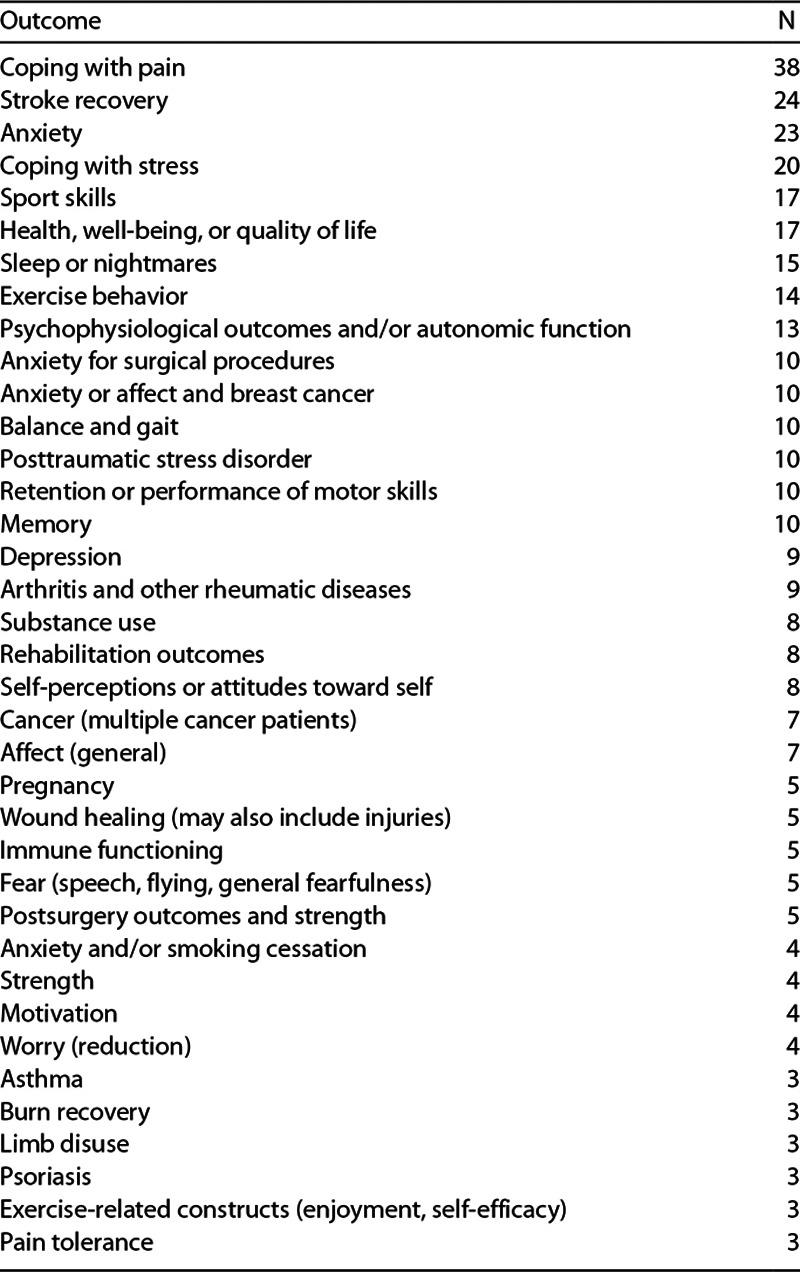
Coded Outcomes

Of the journals shown in Table 1, the studies represented the disciplines of cognitive, clinical, and sport and exercise psychology (n = 7); rehabilitation sciences, including physical medicine, physical therapy, and occupational therapy (n = 5); nursing (n=2) and complementary and alternative medicine (CAM: n = 2).

Table 2 shows the outcomes reported in the studies reviewed. A recurrent theme of research was on psychological outcomes and processes that included studies focused on coping with pain, anxiety or stress, well-being/quality of life, sleep or nightmares, and self-perceptions (e.g., self-efficacy). Guided imagery researchers also tested outcomes related to rehabilitation from stroke, sport skills, exercise behavior, constructs that support exercise behavior (e.g., self-efficacy, motivation), and motor control (e.g., balance and gait). Disease processes and outcomes included posttraumatic stress disorder, a range of cancer sites, immune functioning, arthritis and other rheumatic diseases, substance abuse, asthma, and psoriasis. Other notable health and disease processes focused on anxiety specific to surgical procedures, anxiety or affect related to breast cancer, blood pressure, bleeding and stress during pregnancy and childbirth, general and specific fear, and emotional reactions during smoking cessation.

### Results Reported in Selected Journals

For purpose 2, the results of 13 studies in the 4 selected journals are shown in Table 3. In these studies, the primary outcomes included 7 studies that focused on physiological outcomes,^[22–28]^ 4 that addressed perceptual outcomes related to pain,^[29–32]^ 1 that addressed psychological outcomes,^[33]^ and 1 focused on quality of life.^[34]^ From a methodological standpoint, 8 studies reported outcomes from participant’s self-reports derived from rating scales or surveys,^[22,24,26,29–33]^ 2 used more objective physiological measures related to blood pressure and blood loss,^[27,28]^ while 3 relied on both survey and physiological measures.^[23,25,34]^ Results from these studies showed that guided imagery resulted in significant changes in the observed outcomes in 10 of the 13 (76.9%) studies that supported the authors’ hypotheses that guided imagery resulted in improved outcomes. Since the study outcomes in these studies were vastly different, a quantitative analysis of these studies would not be appropriate.

**TABLE 3.**
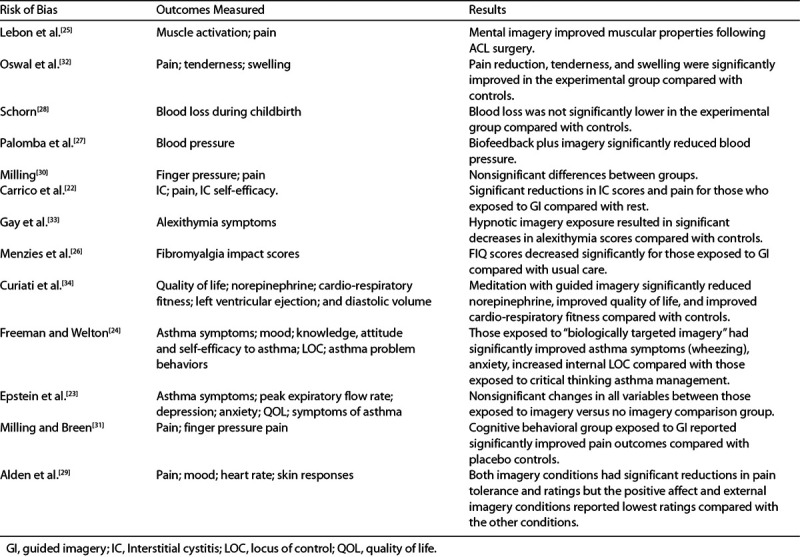
Results in Studies from Selected Journals

### Consultation with Clinicians

Individuals from 15 of the 73 WV PBRN clinical sites offered feedback based on our request (21%). Of these, 11 (73.3%) were coded as clinicians expressing positive views about the potential for using guided imagery in clinical practice, 3 (20.0%) were unsure, and 1 (6.7%) expressed negative views. All responses to the second open-ended survey question about barriers and facilitators regarding the use of guided imagery in clinical practice focused on barriers. These included lack of time, knowledge, training, patient acceptance or skepticism, and concerns about reimbursement for these services. Detailed responses from the clinical respondents are shown in Supplementary File 3 (see **Supplemental Digital Content 3**, http://links.lww.com/PP9/A2).

### Risk of Bias

**Fig. 1.**
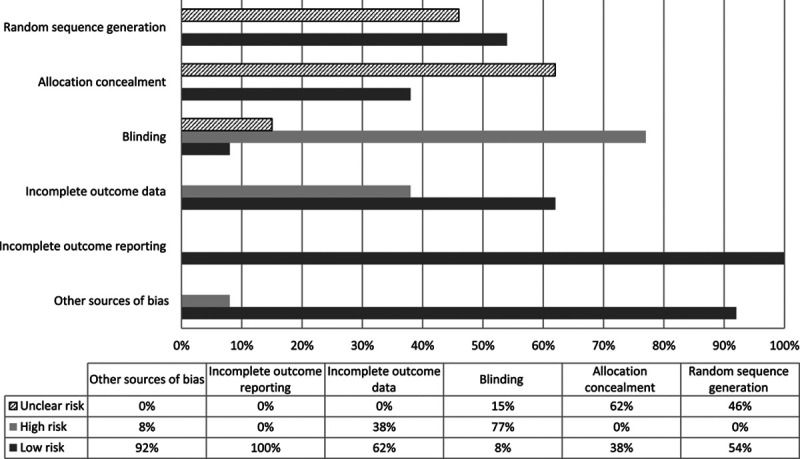
Risk of bias assessments across all studies

Table 4 and Figure 1 show the risk of bias for the 13 selected studies from 4 complementary and integrative health journals. As shown, a large percentage of the studies were coded as being at an unclear or low risk of bias. For sequence generation, 6 studies (46%) were coded as unclear risk,^[25,27,29,30,33,34]^ whereas the other 7 (54%) were considered low risk.^[22–24,26,28,31,32]^ With respect to allocation concealment, 8 studies (62%) were coded as being at an unclear risk of bias,^[23-25,28-30,33,34]^ whereas the remaining 5 (38%) were considered low risk.^[22,26,27,31,32]^ For blinding, 10 studies (77%) were coded as high risk of bias,^[22,23,25,27–30,32–34]^ 2 (15%) were unclear,^[24,26]^ and 1 (8%) was classified as low risk.^[31]^ With regard to incomplete outcome data, 8 studies (62%) were coded as low risk of bias,^[25–31,34]^ while the remaining 5 (38%) were coded as high risk.^[22–24,32,33]^ For incomplete outcome reporting, all 13 studies were coded as low risk of bias.^[22–34]^ For other sources of bias, 12 studies (92%) were coded as being at a low risk of bias,^[22–27,29–34]^ while 1 was at high (8%) risk of bias.^[28]^ The 1 study coded as having high risk of bias for other sources was because the intervention agent was also the first author of the article who interacted directly with research participants.^[28]^ Finally, our own bias assessment focused on blinding study personnel, participants, and both found that 9 studies did not use any blinding procedures^[23–26,28,29,33,34]^ and 5 reported blinding the experimenters only.^[22,27,30–32]^

**TABLE 4.**
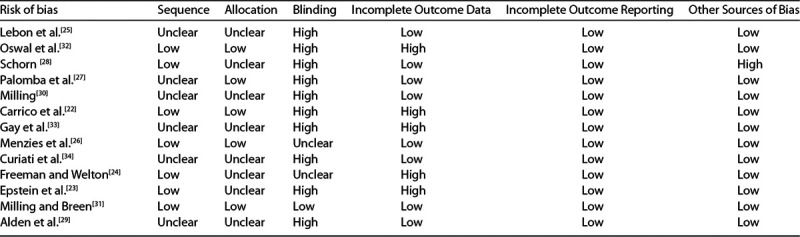
Risk of Bias

## DISCUSSION

The purpose of the current study was to use the scoping review approach to examine the various health outcomes in RCTs in which guided imagery was used as an intervention. Our overall findings suggest that the scientific study of guided imagery includes researchers from diverse disciplines who employ RCT methods to evaluate the effects of this cognitive technique in the treatment of a wide range of disease and health outcomes. Psychologists, sport scientists, medical researchers, rehabilitation specialists, nursing, and other medical professionals all have active lines of scientific inquiry that have tested guided imagery as a primary or tertiary intervention. Not surprisingly, a large number of the studies reviewed were published in psychology journals and focused on stress, affect, and other psychological states. This is likely due to the long history by psychologists using guided imagery for the treatment of affective disorders that include, but are not limited to, generalized anxiety disorder,^[35]^ anger,^[36]^ and posttraumatic stress disorder.^[37]^ Our review also demonstrated that this cognitive technique can be used as a tertiary treatment to alleviate stress and anxiety associated with pregnancy, asthma, surgical, and dental procedures, as well as the treatment of several forms of cancer by nurses or other medical personnel. Sport scientists and rehabilitation specialists have tested the impact of guided imagery on strength, endurance, balance, gait, motor control, and functional tasks of daily living.

A majority of the studies reviewed from the selected integrative health journals, with certain methodological qualifiers, showed positive outcomes related to physical, psychological, and functional changes. Although the results justify future quantitative reviews of the literature and continued research on this topic generally, future interventionists should address the reporting issues and sources of bias discussed in this study. It may be difficult, if not impossible, to eliminate blinding as a source of bias with participants in future RCTs since participants would probably realize that they were assigned to a guided imagery condition. However, blinding study personnel is feasible and should be conducted. Addressing incomplete outcome data as a source of bias may involve finding creative ways to increase retention in future trials. This continued challenge in social and behavioral research is likely compounded in the study of guided imagery because some individuals may be skeptical about the intent or impact of this cognitive technique.

One general observation about these findings is that most of the studies reviewed tested the impact of guided imagery on discrete events or acute symptoms while a considerably smaller number of authors focused on behavior change to address smoking cessation, dietary behavior, and physical activity. Given this apparent shortfall, it would appear plausible to suggest that the use of guided imagery to help individuals regulate behavioral risk factors could be an important future research direction, given the need to address concerns related to chronic disease. Guided imagery could prove to be a valuable intervention strategy because this technique can be delivered using Web- and telephone-based methods while emerging research is testing its suitability using mobile applications.^[38]^ Population-based research testing the efficacy and effectiveness of guided imagery on health behaviors such as diet, exercise, and smoking cessation could yield important insights into its usefulness for public health practitioners in the prevention and/or management of chronic disease.

The final purpose of this study was to gain practitioner feedback about possible barriers to implementing guided imagery in clinical practice. Results indicated that clinicians may be hesitant to implement guided imagery interventions due to time constraints, lack of insurance reimbursement, and perceived lack of expertise. These concerns may require guided imagery practitioners to consider intervention options outside the clinic such as telephone and mobile health applications. As indicated by one of the practitioners in the PBRN, guided imagery could be implemented by clinicians trained in behavioral medicine and this is probably the most logical fit in a clinical setting.

### Study Shortcomings and Future Research

One important shortcoming of this scoping review was lack of quantitative assessments of study outcomes. Specifically, the coded studies were not meta-analyzed because of the diverse outcomes examined and methodological heterogeneity between studies. Another shortcoming relates to the subjective nature of the coding process since many of the study outcomes involved conceptually similar psychological outcomes (i.e., stress and anxiety) as part of complex treatments for medical and disease conditions. However, the purposes here were to document the outcomes addressed by guided imagery researchers to stimulate future intervention research and/or quantitative reviews. Finally, since representatives from only 21% of the WV PBRN sites responded with feedback about our findings, it is possible that more detailed information could be obtained about the use of guided imagery in clinical settings in future studies.

In conclusion, this scoping review demonstrated that guided imagery is a multidisciplinary area of inquiry represented by researchers in psychology, medicine, nursing, rehabilitation, and the sport sciences, among others. We documented the range of health outcomes and disease processes studied by imagery researchers using RCT methods and the journals where this work was published. Also assessed were outcomes observed in selected integrative health journals along with findings from risk of bias assessments. Guided imagery is a common primary and secondary intervention strategy that offers great potential for future research and application.

## Acknowledgments

GA Kelley was partially funded by the National Institute of General Medical Sciences of the National Institutes of Health under award number U54GM104942. The content is solely the responsibility of the authors and does not necessarily represent the official views of the National Institutes of Health. Peter Giacobbi Jr is funded by the West Virginia Prevention Research Center by Cooperative Agreement Number 1-U48-DP-005004 from the Centers for Disease Control and Prevention. The findings and conclusions in this article are those of the authors and do not necessarily represent the official position of the Centers for Disease Control and Prevention.

## Disclosure

The authors have no financial interest to declare in relation to the content of this article. The Article Processing Charge was paid for by Progress in Preventive Medicine at the discretion of the Editor-in-Chief.

## Supplementary Material

## References

[1] Clarke TC Black LI Stussman BJ *Trends in the Use of Complementary Health Approaches Among Adults: United States, 2002–2012*. 2015 Hyattsville, MD: National Center for Health Statistics; PMC4573565 25671660

[2] Mental Imagery. Metaphysics Research Lab, Center for the Study of Language and Information, Stanford University; 2014 Available at http://plato.stanford.edu/archives/fall2014/entries/mental-imagery/.

[3] Jensen MP Psychosocial approaches to pain management: an organizational framework. Pain. 2011;152:717 725. 21168972 10.1016/j.pain.2010.09.002

[4] Rich TA Pfister R Alton J Assessment of cardiovascular parameters during meditation with mental targeting in varsity swimmers. Evid Based Complement Alternat Med. 2016;2016:7923234. 26981142 10.1155/2016/7923234 PMC4766321

[5] Feltz DL Landers DM The effects of mental practice on motor skill learning and performance: a meta-analysis. J Sport Psychol. 1983;5(1):25 57.

[6] Fernandez E Turk DC The utility of cognitive coping strategies for altering pain perception: a meta-analysis. Pain. 1989;38:123 135. 2674861 10.1016/0304-3959(89)90230-3

[7] Van Kuiken D A meta-analysis of the effect of guided imagery practice on outcomes. J Holist Nurs. 2004;22:164 179. 15154991 10.1177/0898010104266066

[8] Jacobsen PB Jim HS Psychosocial interventions for anxiety and depression in adult cancer patients: achievements and challenges. CA Cancer J Clin. 2008;58:214 230. 18558664 10.3322/CA.2008.0003

[9] Chan CK Cameron LD Promoting physical activity with goal-oriented mental imagery: a randomized controlled trial. J Behav Med. 2012;35:347 363. 21695405 10.1007/s10865-011-9360-6

[10] Duncan LR Hall CR Wilson PM The use of a mental imagery intervention to enhance integrated regulation for exercise among women commencing an exercise program. Motiv Emot. 2012;36:452 464.

[11] Missbach B Florack A Weissmann L Mental imagery interventions reduce subsequent food intake only when self-regulatory resources are available. Front Psychol. 2014;5:1391. 25506337 10.3389/fpsyg.2014.01391 PMC4246674

[12] Kemps E Tiggemann M A role for mental imagery in the experience and reduction of food cravings. Front Psychiatry. 2014;5:193. 25610404 10.3389/fpsyt.2014.00193 PMC4284995

[13] Morewedge CK Huh YE Vosgerau J Thought for food: imagined consumption reduces actual consumption. Science. 2010;330:1530 1533. 21148388 10.1126/science.1195701

[14] Bhutani GE Looking after me looking after you: using positive cognitive behavioural techniques to improve emotional well-being. Cogn Behav Ther. 2015;8:110 118.

[15] Bigham E McDannel L Luciano I Effect of a brief guided imagery on stress. Biofeedback. 2014;42(1):28 35.

[16] Kosslyn SM Thompson WL Ganis G. *The Case for Mental Imagery*. 2006 New York, N.Y.: Oxford University Press;

[17] NCCAM. Strategic plan 2011–2015. 2011 Available at http://nccam.nih.gov/about/plans/2011.

[18] Armstrong R Hall BJ Doyle J ‘Scoping the scope’ of a cochrane review. J Public Health. 2011;33(1):147 150. 10.1093/pubmed/fdr015 21345890

[19] Levac D Colquhoun H O’Brien KK Scoping studies: advancing the methodology. Implement Sci. 2010;5:69. 20854677 10.1186/1748-5908-5-69 PMC2954944

[20] Giacobbi PR Jr Stabler ME Stewart J Guided imagery for arthritis and other rheumatic diseases: a systematic review of randomized controlled trials. Pain Manag Nurs. 2015;16:792 803. 26174438 10.1016/j.pmn.2015.01.003 PMC4605831

[21] Higgins JPT Green S. *Cochrane Handbooks for Reviews of Interventions*. 2009 Chichester, West Sussex, England: The Cochrane Collaboration and John Wiley & Sons, Ltd;

[22] Carrico DJ Peters KM Diokno AC Guided imagery for women with interstitial cystitis: results of a prospective, randomized controlled pilot study. J Altern Complement Med. 2008;14:53 60. 18199015 10.1089/acm.2007.7070

[23] Epstein GN Halper JP Barrett EA A pilot study of mind-body changes in adults with asthma who practice mental imagery. Altern Ther Health Med. 2004;10:66 71. 15285276

[24] Freeman LW Welton D Effects of imagery, critical thinking, and asthma education on symptoms and mood state in adult asthma patients: a pilot study. J Altern Complement Med. 2005;11:57 68. 15750364 10.1089/acm.2005.11.57

[25] Lebon F Guillot A Collet C Increased muscle activation following motor imagery during the rehabilitation of the anterior cruciate ligament. Appl Psychophysiol Biofeedback. 2012;37:45 51. 22127572 10.1007/s10484-011-9175-9

[26] Menzies V Taylor AG Bourguignon C Effects of guided imagery on outcomes of pain, functional status, and self-efficacy in persons diagnosed with fibromyalgia. J Altern Complement Med. 2006;12:23 30. 16494565 10.1089/acm.2006.12.23 PMC3712642

[27] Palomba D Ghisi M Scozzari S Biofeedback-assisted cardiovascular control in hypertensives exposed to emotional stress: a pilot study. Appl Psychophysiol Biofeedback. 2011;36:185 192. 21656149 10.1007/s10484-011-9160-3

[28] Schorn MN The effect of guided imagery on the third stage of labor: a pilot study. J Altern Complement Med. 2009;15:863 870. 19678776 10.1089/acm.2008.0567

[29] Alden AL Dale JA DeGood DE Interactive effects of the affect quality and directional focus of mental imagery on pain analgesia. Appl Psychophysiol Biofeedback. 2001;26:117 126. 11480162 10.1023/a:1011387122804

[30] Milling LS Response expectancies: a psychological mechanism of suggested and placebo analgesia. Contemporary Hypnosis (John Wiley & Sons, Inc). 2009;26(2):93 110.

[31] Milling LS Breen A Mediation and moderation of hypnotic and cognitive-behavioural pain reduction. Contemporary Hypnosis (John Wiley & Sons, Inc). 2003;20(2):81 97.

[32] Oswal P Nagarathna R Ebnezar J The effect of add-on yogic prana energization technique (YPET) on healing of fresh fractures: a randomized control study. J Altern Complement Med. 2011;17:253 258. 21417810 10.1089/acm.2010.0001

[33] Gay M-C Hanin D Luminet O Effectiveness of an hypnotic imagery intervention on reducing alexithymia. Contemporary Hypnosis (John Wiley & Sons, Inc). 2008;25(1):1 13.

[34] Curiati JA Bocchi E Freire JO Meditation reduces sympathetic activation and improves the quality of life in elderly patients with optimally treated heart failure: a prospective randomized study. J Altern Complement Med. 2005;11:465 472. 15992231 10.1089/acm.2005.11.465

[35] Borkovec TD Newman MG Pincus AL A component analysis of cognitive-behavioral therapy for generalized anxiety disorder and the role of interpersonal problems. J Consult Clin Psychol. 2002;70:288 298. 11952187

[36] Beck R Fernandez E Cognitive-behavioral therapy in the treatment of anger: a meta-analysis. Cogn Ther Res. 1998;22(1):63 74.

[37] Foa EB Rothbaum BO. *Treating the Trauma of Rape: Cognitive-Behavioral Therapy for PTSD*. 1998 New York, N.Y.: The Guilford Press;

[38] Giacobbi P Jr Hingle M Johnson T See me smoke-free: protocol for a research study to develop and test the feasibility of an mHealth App for women to address smoking, diet, and physical activity. JMIR Res Protoc. 2016;5:e12. 26795257 10.2196/resprot.5126 PMC4742619

