# Supplementary material for: A Scoping Review of Health Outcomes Examined in Randomized Controlled Trials Using Guided Imagery
Source: Prog Prev Med (N Y). 2017 Dec 11;2(7):e0010. doi: 10.1097/pp9.0000000000000010 (PMC5812272; doi:10.1097/pp9.0000000000000010)
Supplement: Supplementary file 1 [file ppm-2-e0010-s001.docx]

Supplementary File

http://search.ebscohost.com.www.libproxy.wvu.edu/login.aspx?direct=true&db=cmedm&bquery=(((((((%26quot%3bmental+imagery%26quot%3b+OR+%26quot%3bguided+imagery%26quot%3b)+OR+((MM+%26quot%3bImagery+(Psychotherapy)%26quot%3b))+OR+((MM+%26quot%3bImagery+(Psychotherapy)%26quot%3b)+AND+TI+(imagery)))+OR+TI+(visualization))+OR+(((MM+%26quot%3bImagery+(Psychotherapy)%26quot%3b))+AND+AB+(Imagery)))+AND+((%26quot%3bTW%26quot%3b+AND+(behavior+OR+behaviour)+AND+chang*)+OR+((MH+%26quot%3bSelf+Efficacy%26quot%3b)+OR+(MH+%26quot%3bSelf+Concept%26quot%3b))+OR+(TX+(%26quot%3bself+efficacy%26quot%3b)+OR+%26quot%3bself+concept%26quot%3b)+OR+((MH+%26quot%3bPatient+Satisfaction%26quot%3b)+OR+(MH+%26quot%3bConsumer+Satisfaction%26quot%3b))+OR+(TX+(%26quot%3bpatient+satisfaction%26quot%3b)+OR+%26quot%3bconsumer+satisfication%26quot%3b)+OR+((MH+%26quot%3bSports%2b%26quot%3b)+OR+(MH+%26quot%3bExercise%2b%26quot%3b)+OR+(MH+%26quot%3brehabilitation%2b%26quot%3b)+OR+(ZU+%26quot%3brehabilitation%26quot%3b)+OR+(MH+%26quot%3bMotor+Activity%26quot%3b))+OR+(TX+(baseball)+OR+basketball+OR+bicycling+OR+Boxing+OR+Football+OR+Rugby+OR+Golf+OR+golfing+OR+Gymnasitics+OR+Hockey+OR+%26quot%3bMartial+Arts%26quot%3b+OR+Karate+OR+%26quot%3bTai+Ji%26quot%3b+OR+Mountaineering+OR+%26quot%3baerobic+exercises%26quot%3b+OR+gait+OR+locomotion+OR+%26quot%3bfine+motor+control%26quot%3b)+OR+(TX+(%26quot%3bracquet+ball%26quot%3b)+OR+%26quot%3bshot+put%26quot%3b+OR+walking+OR+swimming+OR+hiking+OR+%26quot%3bresistance+training%26quot%3b+OR+exercise+OR+%26quot%3bweight+training%26quot%3b+OR+%26quot%3bweight+lifting%26quot%3b+OR+dance+OR+%26quot%3bresistance+exercise%26quot%3b+OR+%26quot%3bresistance+training%26quot%3b)+OR+(TX+(racquetball)+OR+racketball+OR+tennis+OR+lacrosse+OR+running+OR+jogging+OR+skating+OR+skiing+OR+soccer+OR+swimming+OR+Diving+OR+Track+OR+decathlon+OR+%26quot%3bshot+put%26quot%3b+OR+volleyball+OR+dancing+OR+walking+OR+%26quot%3bstrength+training%26quot%3b+OR+%26quot%3bweight+lifting%26quot%3b+OR+%26quot%3bweight+training%26quot%3b+OR+javelin+OR+wrestling+OR+s
