# Supplementary material for: A Scoping Review of Health Outcomes Examined in Randomized Controlled Trials Using Guided Imagery
Source: Prog Prev Med (N Y). 2017 Dec 11;2(7):e0010. doi: 10.1097/pp9.0000000000000010 (PMC5812272; doi:10.1097/pp9.0000000000000010)
Supplement: Supplementary file 2 [file ppm-2-e0010-s002.docx]

“Guided imagery is the controlled visualization in one's mind of specific thoughts, feelings, or experiences. People regularly use imagery to plan events or learn new skills. Guided imagery typically involves scripts or audio files and is often used in clinical settings to help patients manage stress or pain.  We reviewed randomized controlled trials (RCTs) that tested the impact of guided imagery on disease and health outcomes. We found 322 RCTs that used guided imagery to address pain, patient preparation for surgical procedures, sport and exercise outcomes, stroke rehabilitation, coping with stress, and hypertension, among other outcomes. We also found that results were positive in 10 of the 13 studies reviewed in integrative health and medical journals. In these studies, significant improvements were observed in pain perceptions, changes in mental health outcomes, hypertension, and improved stroke rehabilitation outcomes in patients often cared for in primary care settings.”

Your responses to the following questions would be greatly appreciated: 1) Please share your general thoughts about the potential use of guided imagery in your clinical practice, and 2) Please describe any potential barriers or facilitators for the use of guided imagery in your clinical practice considering that this cognitive technique can be delivered remotely or in-person.
